# Integrated Multi-Omics Analysis Reveals the Role of Resveratrol in Regulating the Intestinal Function of *Megalobrama amblycephala* via m^6^A Methylation

**DOI:** 10.3390/ijms26178587

**Published:** 2025-09-03

**Authors:** Zhengyan Gu, Qiaoqiao Mu, Linjie Qian, Yan Lin, Wenqiang Jiang, Siyue Lu, Linghong Miao, Xianping Ge

**Affiliations:** 1 Wuxi Fisheries College, Nanjing Agricultural University, Wuxi 214081, China; guzhengyan@ffrc.cn (Z.G.); qiaoqiao72021@163.com (Q.M.); qianlinjiejie@gmail.com (L.Q.); 2 Key Laboratory of Freshwater Fisheries and Germplasm Resources Utilization, Ministry of Agriculture and Rural Affairs, Freshwater Fisheries Research Center, Chinese Academy of Fishery Sciences, Wuxi 214081, China; liny@ffrc.cn (Y.L.); jiangwenqiang@ffrc.cn (W.J.); lusiyue@ffrc.cn (S.L.)

**Keywords:** resveratrol, intestinal barrier function, m^6^A methylation, endoplasmic reticulum stress, high lipid metabolism

## Abstract

Resveratrol (RES), a natural polyphenol with lipid metabolism-regulating properties, also demonstrates remarkable efficacy in strengthening intestinal barrier integrity. In order to elucidate the mechanism by which RES ameliorates intestinal damage and lipid metabolism disturbances in *Megalobrama amblycephala* under a high-fat (HF) diet, a conventional diet (CON), an HF diet (HF), or an HF diet supplemented with 0.6, 3, or 6 g/kg RES (HF + 0.06%, 0.3%, or 0.6% RES) was fed to fish. After 8 weeks, RES supplementation in the HF diet significantly improved the growth performance and alleviated hepatic lipid deposition. Microbiota profiling revealed RES improved intestinal barrier function by reducing α-diversity, Actinobacteria and *Bosea* abundances, and enriching Firmicutes abundance. RES also maintained the integrity of the intestinal physical barrier and inhibited the inflammatory response. MeRIP-seq analysis indicated that RES modulated intestinal mRNA m^6^A methylation by upregulating methyltransferase-like 3 (*mettl3*) and downregulating fat mass and obesity-associated gene (*fto*) and Alk B homolog 5 (*alkbh5*). Combined RNA-seq and MeRIP-seq data revealed that RES alleviated endoplasmic reticulum stress (ERS) by upregulating the m^6^A methylation and gene level of heat shock protein 70 (*hsp70*). Correlation analyses identified significant associations between intestinal microbiota composition and ERS, tight junction, and inflammation. In summary, RES ameliorates lipid dysregulation via a synergistic mechanism involving intestinal microbiota, m^6^A modification, ERS, barrier function, and inflammatory response.

## 1. Introduction

As essential nutrients for fish growth, lipids play a pivotal role in exerting a protein-sparing effect within feed formulations. Researches have indicated that appropriate modulation of dietary lipid levels can substantially improve protein efficiency and deposition [1]. However, this nutritional strategy is constrained by a critical dose-dependent threshold. Prolonged high-fat (HF) diet intake readily leads to excessive lipid accumulation, contributing to metabolic dysfunction and increased mortality, ultimately resulting in economic losses in aquaculture [2]. Studies have shown that fish exhibit a considerably lower tolerance to excess lipids in the diet than mammals [3]. Chronic feeding of aquatic animals with HF diets not only compromises intestinal barrier integrity by downregulating tight junction proteins and increasing intestinal permeability, but also triggers microbiota dysbiosis, excessive secretion of inflammatory cytokines, and endotoxin translocation. These effects exacerbate systemic metabolic abnormalities through the intestine–liver axis network [4,5]. The functional integrity of the intestine is fundamental for maintaining fish health as the first line of defense against pathogens and a vital regulator of internal homeostasis [6]. Accordingly, the identification of safe and effective feed additives to counteract HF diet-induced intestinal damage and metabolic dysregulation has emerged as a key focus in aquatic nutrition research.

Resveratrol (RES), a naturally occurring plant-derived polyphenol possesses high medicinal value. It possesses diverse functions such as regulating lipid metabolism, resisting oxidative stress, and suppressing inflammatory responses [7,8,9]. These broad bioactivities also enable RES to exert significant therapeutic effects in modulating intestinal functions and enhancing intestinal barrier integrity. In red tilapia (*Oreochromis niloticus*), RES can repair HF diet-induced intestinal mucosal damage by increasing the goblet cell density and mucus secretion [10]. Studies on koi carp (*Cyprinus carpio*) demonstrate that RES enhances disease resistance by remodeling the composition of intestinal microbiota, such as raising the abundance of *Lactobacillus* [11]. Notably, recent research has shown that RES acts as a regulator of N^6^-methyladenosine (m^6^A) methyltransferase-like 3 (METTL3), influencing RNA epigenetic modification patterns to modulate the stability and translational efficiency of peroxisome proliferator-activated receptor alpha (*ppar-α*) in mice [12]. The above research indicates that these biological activities of RES are closely associated with the regulation of the mRNA m^6^A methylation level.

In eukaryotes, m^6^A methylation represents the most prevalent form of RNA modification. It precisely regulates the post-transcriptional gene expression via the synergistic role of methyltransferases, demethylases, and m^6^A-binding proteins, thus modulating multiple biological functions [13]. The study indicates that dietary RES supplementation enhances intestinal functionality and antioxidant capacity by reducing m^6^A methylation levels of heme oxygenase-1 (*ho-1*) and tight junction protein mRNAs, improving piglets’ growth performance [14]. Moreover, RES alleviates hepatic steatosis in obese mice by inhibiting the activity of the demethylase fat mass and obesity-associated (FTO) protein, thus upregulating the expression of hepatic genes related to lipid metabolism [12]. Recent evidence suggests that m^6^A modifications may serve as a key epigenetic target through which RES modulates intestinal homeostasis and lipid metabolism. However, the biological impact of RES-mediated m^6^A modifications on intestinal function in fish remains undefined, and a significant gap persists in understanding its regulatory role within the ‘nutrition–epigenetics–intestinal function’ axis.

*Megalobrama amblyocephala*, also known as Wuchang bream, belongs to Cypriniformes, Cyprinidae, and *Megalobrama*. As a major freshwater species of high economic value, *M. amblyocephala* occupies a prominent position in Chinese aquaculture. However, the emergence of metabolic syndrome associated with prolonged HF diet administration under intensive farming conditions has become increasingly evident, posing a significant barrier to industrial development [15,16]. Therefore, this study aimed to elucidate the epigenetic regulatory function of RES in reducing growth retardation and intestinal damage, while enhancing lipid metabolism via the intestine–liver axis in HF diet-fed juveniles. The results provide a theoretical basis for incorporating RES into aquafeed and reveal a novel mechanism by which m^6^A methylation regulates in nutrient metabolism, thus offering innovative approaches for precision nutrition and sustainable aquaculture practices.

## 2. Results

### 2.1. Effects of RES on the Growth Performance and Lipid Metabolism of Juveniles Fed with an HF Diet

**Figure 1:**
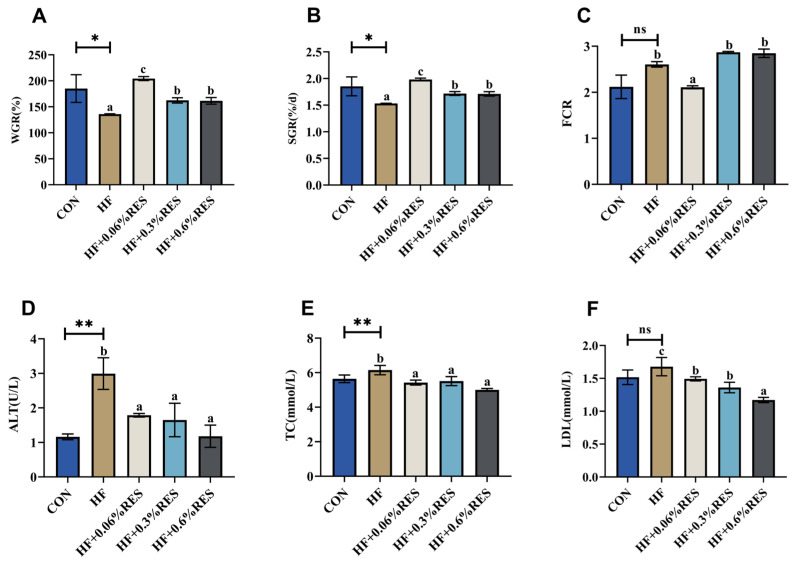

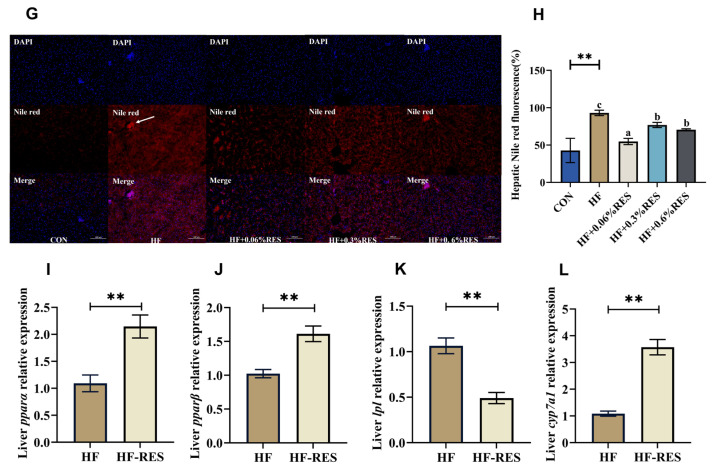
Effects of RES on the growth performance and lipid metabolism of juveniles fed with an HF diet. (**A**–**C**) Growth performance indicators: weight gain rate (WGR), specific growth rate (SGR), and feed conversion ratio (FCR). (**D**–**F**) Plasma biochemical parameters: alanine aminotransferase (ALT), total cholesterol (TC), and low-density lipoprotein (LDL). (**G**,**H**) Nile Red staining of hepatic tissues; red fluorescence (white arrows) indicates lipid droplets, and blue fluorescence marks nuclei. (**I**–**L**) Hepatic mRNA levels of peroxisome proliferator-activated receptor alpha (*ppar-α*), peroxisome proliferator-activated receptor beta (*ppar-β*), lipoprotein lipase (*lpl*), and cholesterol 7-alpha hydroxylase (*cyp7a1*). HF-RES group stands for HF + 0.06% RES group. Data are expressed as mean ± standard error mean (SEM). Significant differences in Independent *t*-test are denoted by asterisks, * *p* < 0.05, ** *p* < 0.01, and ns indicates no significant difference. While differences in Duncan’s test are labeled with distinct lowercase letters (*p* < 0.05).

After the feeding trial (8 weeks), juvenile *M. amblycephala* growth performance indices are shown in Figure 1A–C. Compared with the CON group, the HF group showed significant reductions in weight gain rate (WGR) and specific growth rate (SGR) (Independent *t*-test, *p* < 0.05). The HF + 0.06%, 0.3%, and 0.6% RES groups showed significant improvements in WGR and SGR relative to the HF group (Duncan’s test, *p* < 0.05). Compared with the HF group, the feed conversion ratio (FCR) in the HF + 0.06% RES group was dramatically decreased (Duncan’s test, *p* < 0.05). Figure 1D–F demonstrates that plasma alanine aminotransferase (ALT) and total cholesterol (TC) concentrations in the HF group were significantly greater than the CON group (Independent *t*-test, *p* < 0.01). Compared with the HF group, plasma ALT, TC, and low-density lipoprotein (LDL) levels in the three RES addition groups were significantly decreased (Duncan’s test, *p* < 0.05). Quantitative analysis using ImageJ software (v1.8.0.112) confirmed that the red area of hepatic lipid droplet was considerably larger in the HF group than in the CON group (Figure 1G,H, Independent *t*-test, *p* < 0.01). Each RES-treated group showed a significant reduction in lipid accumulation compared with the HF group (Figure 1G,H, Duncan’s test, *p* < 0.05). Among the RES doses, 0.06% RES yielded significant positive effects. Therefore, gene expression was analyzed between HF and HF + 0.06% RES (HF-RES) groups. Hepatic gene levels of *ppar-α*, peroxisome proliferator-activated receptor beta (*ppar-β*), and cholesterol 7-alpha hydroxylase (*cyp7a1*) were significantly upregulated, whereas lipoprotein lipase (*lpl*) expression was downregulated in the HF-RES group compared with the HF group (*p* < 0.01; Figure 1I–L).

### 2.2. Intestinal Microbiota Analysis

**Figure 2:**
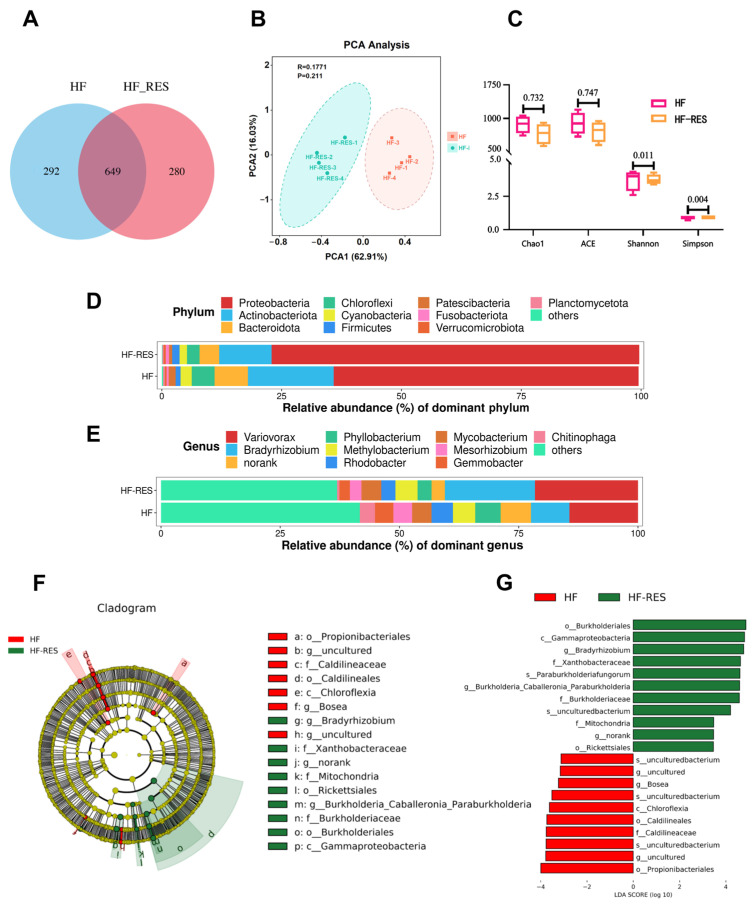
Intestinal microbiota analysis of juveniles *M. amblycephala*. HF-RES group stands for HF + 0.06% RES group. (**A**) Venn diagram of OTUs in the intestinal microbiota of juvenile *M. amblycephala*. (**B**) PCA of intestinal microbiota in juvenile *M. amblycephala*. (**C**) Alpha diversity indices (Shannon, Simpson, Chao1, and ACE) of intestinal microbiota; values above the error bars indicate *p*-values. (**D**) Differential microbiota at the phylum classification. (**E**) Differential microbiota at genus classification. (**F**) Evolutionary branching diagram. (**G**) Histogram of LDA scores distribution (LDA > 2). LEfSe was performed using the OmicStudio tools at https://www.omicstudio.cn/tool/ accessed on 20 May 2025.

The hind intestinal contents from the HF group and the HF + 0.06% RES group (HF-RES group) were selected for intestinal microbiota profiling. Operational taxonomic units (OTUs) were clustered at a similarity threshold of ≥97% using the RDP classifier Bayesian algorithm. A total of 1221 OTUs were identified across the eight samples, classified into 25 phyla, 60 classes, 152 orders, 269 families, 480 genera, and 631 species. Venn diagram analysis (Figure 2A) revealed 649 OTUs shared between the HF and HF-RES groups, with 292 OTUs unique to the HF group and 280 unique to the HF-RES group after the 8-week dietary intervention. Principal component analysis (PCA) (Figure 2B) of intestinal microbiota showed a significant separation between the HF and HF-RES groups, with the first two primary components responsible for 62.91% (PCA1) and 16.03% (PCA2) of variation between groups, respectively. Alpha diversity analysis revealed significantly reduced Shannon and Simpson indices in the HF-RES group compared with the HF group (*p* < 0.05), while Chao1 and ACE indices showed no significant differences (*p* > 0.05) (Figure 2C).

At the phylum level (Figure 2D), the HF-RES group displayed increased relative abundances of Proteobacteria and Firmicutes, whereas Actinobacteriota and Bacteroidota were more abundant in the HF group. At the genus level (Figure 2E), higher relative abundances of *Variovorax* and *Bradyrhizobium* were observed in the HF-RES group, while *Phyllobacterium*, *Mycobacterium*, and *Chitinophaga* showed higher enrichment levels in the HF group.

Based on LEfSe (Linear Discriminant Analysis Effect Size) analysis (Figure 2F), the HF-RES group showed a higher number of differentially abundant microbial taxa than the HF group. As shown in Figure 2G, taxa with linear discriminant analysis (LDA) scores > 2 in the HF-RES group included *Gammaproteobacteria*, *Burkholderiales*, *Mitochondria*, and *Rickettsiales*, while *Propionibacteriales*, *Caldilineaceae*, *Chloroflexia*, and *Bosea* were predominant in the HF group.

### 2.3. Role of RES in Maintaining Intestinal Barrier Health in Juvenile M. amblycephala Fed with an HF Diet

**Figure 3:**
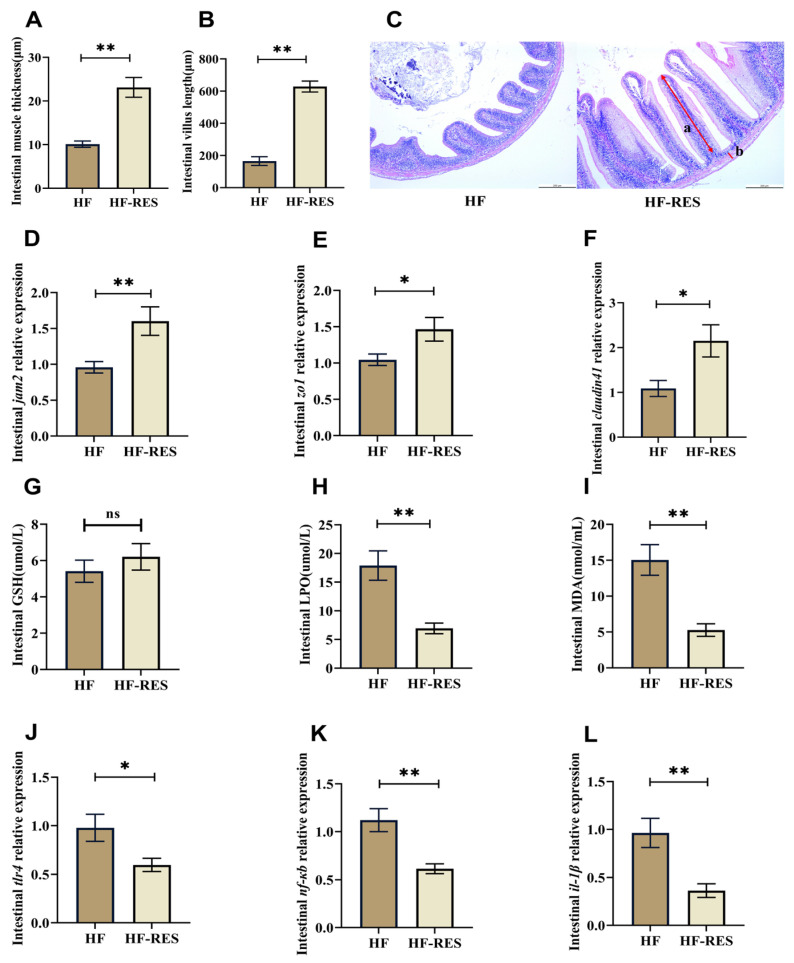
RES role in maintaining intestinal barrier health in juveniles *M. amblycephala* fed with an HF diet. HF-RES group stands for HF + 0.06% RES group. (**A**–**C**) H&E staining of the intestinal tissues, (a) Villus length, (b) muscular layer thickness. (**D**–**F**) Gene expression levels of junctional adhesion molecule 2 (*jam2*), zonula occludens 1 (*zo1*), and *claudin41*. (**G**–**I**) Glutathione (GSH), lipid peroxide (LPO), and malondialdehyde (MDA) levels as indicators of intestinal antioxidant status. (**J**–**L**) Expression levels of inflammatory markers: toll-like receptor 4 (*tlr4*), nuclear factor kappa-B (*nf-κb*), and interleukin-1beta (*il-1β*). Data are presented as mean ± Standard Error Mean (SEM). Significant differences determined by Independent *t*-test are indicated by asterisks: * *p* < 0.05, ** *p* < 0.01, and ns indicates no significant difference.

Intestinal tissues from the HF group and the HF + 0.06% RES group (HF-RES group) were selected to investigate the protective effect of RES against the intestinal injuries and on barrier health of HF diet-fed juveniles. The Hematoxylin and Eosin (H&E) staining results of the intestinal tissues (Figure 3A–C) revealed that the HF-RES group exhibited a clear increase in intestinal muscle thickness and villus length compared with the HF group (*p* < 0.01). Analysis of antioxidant indicators showed that 0.06% RES supplementation in the HF diet markedly reduced the levels of lipid peroxide (LPO) and malondialdehyde (MDA) in the intestinal tissues (Figure 3H,I; *p* < 0.01). As illustrated in Figure 3D–F,J–L, compared with the HF group, the HF-RES group displayed upregulated expression levels of junctional adhesion molecule 2 (*jam2*) (*p* < 0.01), zonula occludens 1 (*zo1*), and *claudin41* (*p* < 0.05), and downregulated expression levels of toll-like receptor 4 (*tlr4*) (*p* < 0.05), nuclear factor kappa-B (*nf-κb*) and interleukin-1beta (*il-1β*) (*p* < 0.01).

### 2.4. MeRIP-seq of the Intestinal Tissues

**Figure 4:**
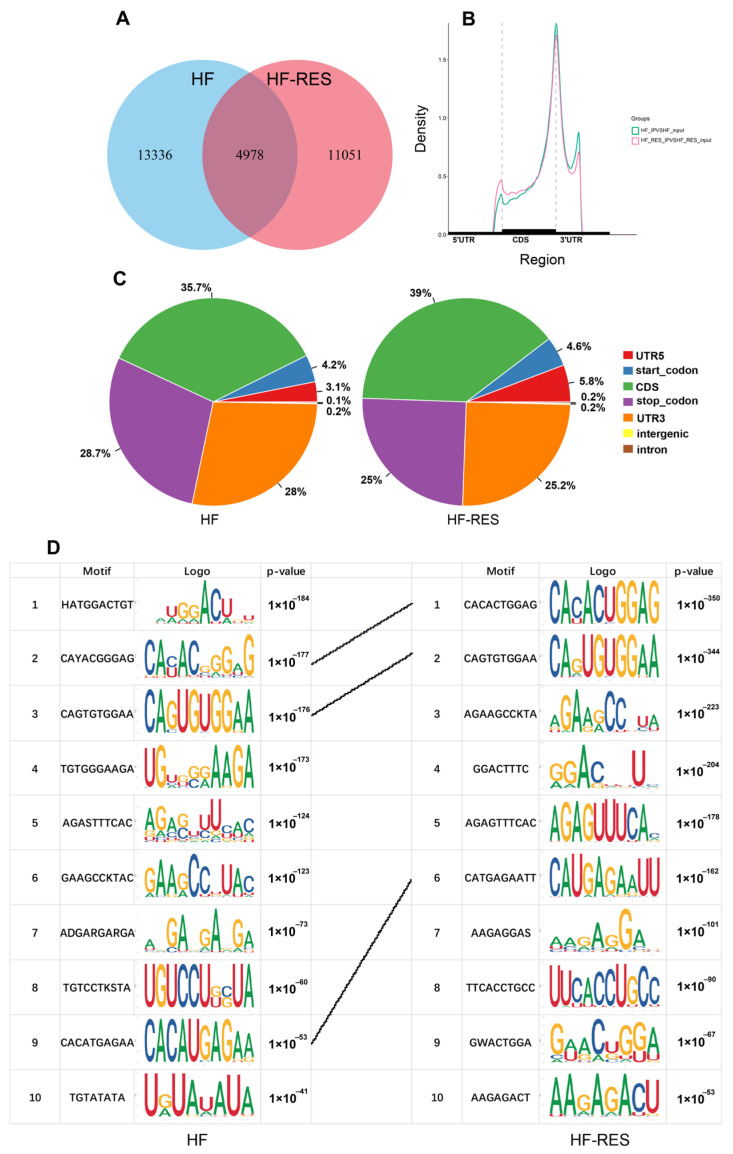
MeRIP-seq analysis of the intestinal tissues. HF-RES group stands for HF + 0.06% RES group. (**A**) Venn diagram of mRNA m^6^A peaks in HF and HF-RES groups. (**B**) Distribution of m^6^A peaks across mRNA CDS, 5′ UTR, and 3′ UTR regions in HF and HF-RES groups. (**C**) Pie charts illustrating the regional distribution of m^6^A peaks in the HF and HF-RES group. (**D**) Sequence motifs enriched in m^6^A-modified region in both groups.

Intestinal tissues from the HF group and the HF + 0.06% RES group (HF-RES group) were selected for MeRIP-seq. The sequencing results of the IP and Input libraries showed that more than 81.97% of the clean data mapped to the *M. amblycephala* reference genome, with preeminent concordance among replicates (Q30 > 93.75%, Q20 > 97.72%) (Tables S1 and S2). A total of 18,314 and 16,029 m^6^A peaks were identified in the HF and HF-RES groups, respectively, with 4978 peaks shared between both groups (Figure 4A). As presented in Figure 4B, m^6^A peaks in both groups were predominantly enriched in coding sequences (CDS), terminal regions of the 5′ untranslated regions (5′ UTRs), and initiation regions of the 3′ untranslated regions (3′ UTRs). According to Figure 4C, the HF group showed m^6^A peak distributions of 35.7% in CDS regions, 28.7% in termination regions, 28% in 3′ UTRs, 4.2% in promoter regions, and 3.1% in 5′ UTRs. In comparison, the HF-RES group showed distributions of 39%, 25%, 25.2%, 4.6%, and 5.8%, respectively. Figure 4D displays the conserved m^6^A motifs identified in both groups, with shared methylation sites indicated by lines, though variations in frequency were observed.

### 2.5. Differential m^6^A Peaks Identification and Functional Analysis in HF and HF-RES Groups

All 20 m^6^A peaks demonstrating the most significant methylation alterations are listed in Table 1. Among these, elevated methylation levels were identified in NYN domain and retroviral integrase containing (*nynrin*), histone H3 family member (*histone H3.v1*), neuraminidase 4 (*neu4*), carcinoembryonic antigen-related cell adhesion molecule 1 (*ceacam1*), OTU deubiquitinase 7A (*otud7a*), interferon regulatory factor 4 (*irf4*), zinc finger protein 184 (*znf184*), slit guidance ligand 2A (*slitla*), solute carrier family 2 member 12 (*slc2a12*), zinc finger protein Y-linked 1 (*zfy1*), bone morphogenetic protein-like A (*bmpla*), cysteine and tyrosine-rich 1 (*cyyr1*), mitochondrial transcription rescue factor 1 (*mtres1*), LOC125253164, nuclear factor erythroid 2-like 1B (*nfe2l1b*), and zinc finger protein 692 (*znf692*). Decreased methylation levels were observed in tripartite motif-containing protein 39 (*trim39*), tetratricopeptide repeat domain 38 (*ttc38*), LOC125260582, and ribonuclease inhibitor (*ri*).

**Figure 5:**
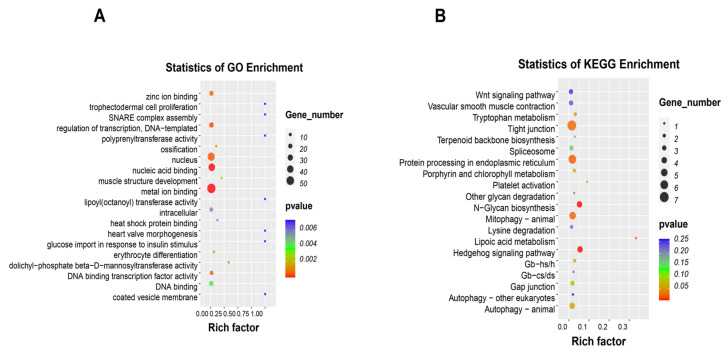
Analysis of GO and KEGG enrichment for differential m^6^A peaks. (**A**) GO enrichment scatter plot. (**B**) KEGG enrichment scatter plot.

Gene Ontology (GO) functional categorization showed categories such as metal ion binding, nucleic acid binding, nucleus, glucose import in response to insulin stimulus, and heat shock protein binding (Figure 5A). Kyoto Encyclopedia of Genes and Genomes (KEGG) analysis further linked these differential peaks to signaling pathways such as Wnt, tight junctions, protein processing in the endoplasmic reticulum, mitophagy–animal, and autophagy–animal (Figure 5B).

### 2.6. Association Analysis Between DEGs and Differential m^6^A Peaks

**Figure 6:**
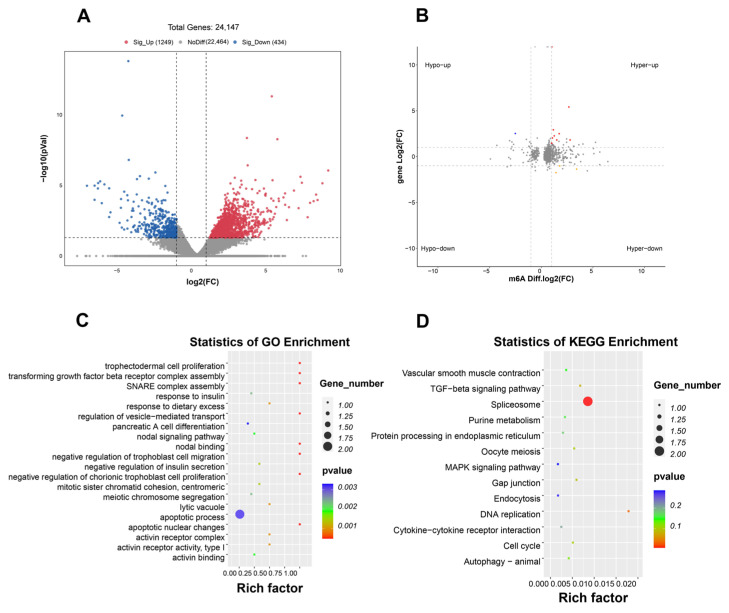
Relationship between DEGs and differential m^6^A peaks. HF-RES group stands for HF + 0.06% RES group. (**A**) DEGs Volcano plot between HF and HF-RES group. (**B**) Four-quadrant plot illustrating the association between DEGs and differential m^6^A peaks. Red represents significant upregulation, blue represents significant downregulation, and gray represents insignificant differences. (**C**) GO enrichment scatter plot for DEGs under an integrated analysis of MeRIP-seq and RNA-seq. (**D**) KEGG enrichment scatter plot for DEGs under an integrated analysis of MeRIP-seq and RNA-seq.

RNA-seq results revealed 1683 differentially expressed genes (DEGs) (|log_2_(fold change) | ≥ 1 and *p* < 0.05) between the HF and HF-RES groups (HF + 0.06% RES group), including 1249 upregulated and 434 downregulated genes (Figure 6A). Integrated analysis of RNA-seq and MeRIP-seq identified 12 genes presenting differential mRNA expressions and m^6^A methylation levels. These genes were divided into four expression patterns (Figure 6B): nine genes including *hsp70*, UV radiation resistance-associated gene (*uvrag*), transmembrane protein 119B (*tmem119b*), protein phosphatase one regulatory subunit 13B, a (*ppp1r13ba*), FK506 binding protein 5 (*fkbp5*), ribosome production factor 2 homolog (*rpf2*), minichromosome maintenance complex component 3 (*mcm3*), structural maintenance of chromosomes 4 (*smc4*), and guanylate cyclase one soluble subunit beta 2 (*gucy1b2*) showed increased m^6^A methylation and upregulated mRNA expression (Hyper-up); mitochondrial transcription rescue factor 1 (*mtres1*) and tumor necrosis factor (ligand) superfamily member 10 (*tnfsf10*) showed elevated m^6^A methylation but reduced mRNA expression (Hyper-down); shugoshin-like 1 (*sgo1*) displayed decreased m^6^A methylation alongside increased mRNA expression (Hypo-up). However, no genes were observed with downregulated m^6^A methylation and reduced mRNA expression (Hypo-down). GO analysis revealed that the 12 genes were primarily enriched in biological processes including apoptotic nuclear changes, apoptotic process, response to dietary excess, negative regulation of insulin secretion, and nodal binding (Figure 6C). KEGG pathway analysis indicated associations with signaling pathways, such as TGF-beta, MAPK, protein processing in the endoplasmic reticulum, gap junction, and autophagy-animal (Figure 6D).

### 2.7. Expression Analysis of Intestinal m^6^A Methylase and Endoplasmic Reticulum Stress-Related Genes

**Figure 7:**
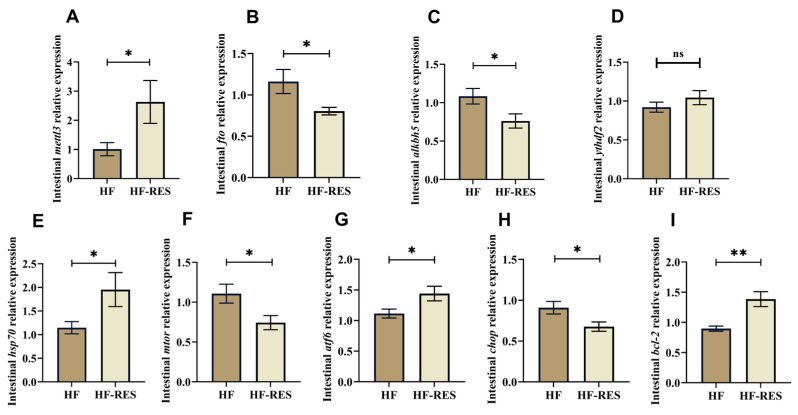
Expression analysis of intestinal m^6^A methylation and ERS-related genes. HF-RES group stands for HF + 0.06% RES group. (**A**–**I**) Intestinal gene expression levels of *mettl3*, *fto*, *alkbh5*, YTH N6-methyladenosine RNA binding protein 2 (*ythdf2*), *hsp70*, mammalian target of rapamycin (*mtor*), activating transcription factor 6 (*atf6*), C/EBP-homologous protein (*chop*) and B-cell lymphoma-2 (*bcl-2*). Data are presented as mean ± standard error mean (SEM). Significant differences determined by Independent *t*-test are indicated by asterisks: * *p* < 0.05, ** *p* < 0.01, and ns indicates no significant difference.

Intestinal tissues from the HF and the HF + 0.06% RES groups (HF-RES group) were selected to analyze the gene levels related to m^6^A methylation and endoplasmic reticulum stress (ERS). Compared with the HF group (Figure 7A–D), *mettl3* expression was significantly elevated, whereas *fto* and Alk B homolog 5 (*alkbh5*) levels were reduced in the HF-RES group (*p* < 0.05), with no significant difference detected in YTH N6-methyladenosine RNA binding protein 2 (*ythdf2*) expression (*p* > 0.05). As depicted in Figure 7E–I, HF-RES group showed significantly increased expression of B-cell lymphoma-2 (*bcl-2*) (*p* < 0.01), heat shock protein 70 (*hsp70*), and activating transcription factor 6 (*atf6*) (*p* < 0.05), while C/EBP-homologous protein (*chop*) and mechanistic target of mammalian target of rapamycin (*mtor*) transcript levels were considerably reduced (*p* < 0.05), indicating modulation of ERS-related gene responses.

### 2.8. Intestinal Microbiota, m^6^A Methylation, Intestinal Barrier, and Lipid Metabolism Correlation Analysis

**Figure 8:**
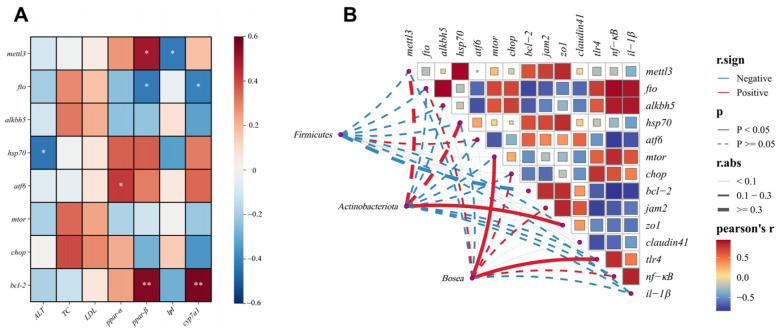
Intestinal microbiota, m^6^A methylation, intestinal barrier, and lipid metabolism correlation analysis. (**A**) Pearson correlation heatmap. Red and blue indicate positive and negative correlations, respectively. Color intensity and square size reflect the strength of associations. * *p* < 0.05, ** *p* < 0.01. (**B**) Combined Pearson correlation heatmap and Mantel test-based network analysis. The network diagram illustrates significant correlations with solid lines (*p* < 0.05); line thickness corresponds to the absolute value of the correlation coefficient.

Intestinal *mettl3* expression level positively correlated with the hepatic *ppar-β* expression level and negatively associated with the *lpl* expression level (*p* < 0.05, Figure 8A). Intestinal *fto* showed inverse correlations with hepatic *ppar-β* and *cyp7a1* (*p* < 0.05). Intestinal *hsp70* negatively associated with plasma ALT level (*p* < 0.05). Intestinal *atf6* was positively associated with hepatic *ppar-α* expression (*p* < 0.05). Intestinal *bcl-2* expression demonstrated strong positive correlations with hepatic *ppar-β* and *cyp7a1* expression levels (*p* < 0.01).

Intestinal microbiota, which played important roles in the HF and the HF-RES groups (HF + 0.06% RES group), were prioritized for correlation analysis to decipher their interplay with genes related to m^6^A methylation, intestinal barrier, and inflammation (Figure 8B). *Bosea* positively correlated with *mtor* and *tlr4* expressions (*p* < 0.05). Actinobacteria were positively associated with *zo1* (*p* < 0.05). *Mettl3* had positive correlations with *hsp70*, *jam2* and *zo1* (*p* < 0.05). *Fto* expression showed a positive regulatory relationship with *tlr4*, *nf-κb,* and *il-1β*, while negatively correlated with *bcl-2* (*p* < 0.05). *Alkbh5* showed positive connections with *chop*, *nf-κb,* and *il-1β*, but a negative correlation with *atf6* (*p* < 0.05). *Hsp70* depicted a regulatory relationship with *zo1* (*p* < 0.05). *Atf6* revealed negative correlations with *il-1β* and *nf-κb* (*p* < 0.05). *Mtor* showed a positive regulatory relationship with *nf-κb* (*p* < 0.05). *Chop* also demonstrated a regulatory relationship with *tlr4* (*p* < 0.05). *Bcl-2* expression was positively related to *jam2* and *zo1* but negatively associated with *il-1β* and *nf-κb* (*p* < 0.05). *Tlr4* demonstrated negative correlations with *jam2* and *zo1* (*p* < 0.05).

## 3. Discussion

In aquaculture, lipids are routinely used as alternative energy sources to partially replace dietary protein, reduce feed costs, and enhance protein utilization efficiency [17]. However, an excessive lipid concentration in the feed may induce adverse physiological responses in fish, including inhibited growth, disrupted lipid metabolism, oxidative stress, and compromised immune function [18]. Soybean oil is rich in polyunsaturated fatty acids (PUFAs), predominantly ω-6 PUFAs [19]. Excessive intake of ω-6 PUFAs can disrupt the ω-6/ω-3 balance, leading to abnormal lipid metabolism and inflammatory responses [20]. Studies on fish and mammals show that soybean oil has been successfully used to establish high-fat-induced injury models [21,22,23]. Similarly, in this experiment, we constructed a high-fat injury model for *M. amblycephala* by adding excessive soybean oil to the diet in this experiment. Previous studies have demonstrated that supplementation of RES in an HF diet significantly alleviates lipid-induced growth suppression in common carp, as reflected by elevated WGR and improved FCR [22]. These findings are consistent with the current results, wherein dietary RES supplementation in an HF diet enhanced WGR and SGR, reduced FCR, and alleviated growth suppression in HF diet-fed juvenile *M. amblycephala*.

Plasma ALT, TC, and LDL contents are crucial indicators for assessing lipid metabolism. The liver, functioning as the central regulator of lipid metabolism, facilitates lipid digestion and absorption through bile acid secretion and controls fatty acid oxidation [24]. CYP7A1 acts as a key enzyme in bile acid biosynthesis, whereas PPARs and LPL are key regulators of lipid oxidation and hydrolysis. In the present study, RES supplementation significantly attenuated HF diet-induced increases in plasma ALT, TC, and LDL levels, evidently upregulated hepatic expressions of *cyp7a1*, *ppar-α*, and *ppar-β*, and suppressed *lpl* gene expression. Based on Nile Red staining, RES reduced hepatic lipid deposition induced by the HF diet in juveniles. Similar results were observed in red tilapia, where dietary RES significantly decreased serum ALT, TC, and LDL levels, downregulated hepatic *lpl* expression, and upregulated *ppar-α* expression [25]. These findings indicate that the regulatory effects of RES on lipid metabolism in fish may be attributed to the high hepatic lipid catabolism. Furthermore, among the tested RES concentrations, 0.06% RES in the HF diet produced a significant therapeutic effect. Thus, a comparative analysis between the HF and HF + 0.06% RES groups (designated as HF-RES group) was undertaken to explore specific mechanisms underlying RES-mediated regulation of intestinal health and lipid metabolism.

The intestinal barrier is a multi-layered defense system that balances the intestinal internal and external environments. Its dynamic functional equilibrium relies on microbial community stability, mucus layer integrity, and immune regulation. In the intestinal biological barrier, RES treatment has been widely reported to enhance the α-diversity of gut microbiota in mice, ameliorating dysbiosis associated with HF dietary intake [26]. However, the present findings demonstrated a divergent outcome, wherein RES supplementation under HF dietary conditions resulted in a reduction in α-diversity of the intestinal microbiota in juvenile *M. amblycephala*, as indicated by decreases in Shannon and Simpson diversity indices. Physiologically, fish possess shorter intestines and simpler microbial compositions compared to mammals, which may lead to differences in the absorption and metabolism of RES, consequently resulting in distinct regulatory effects on the intestinal microbiota [27]. Previous studies in diabetic mouse models have suggested that RES facilitates the production of intestinal short-chain fatty acids (SCFAs), which may indirectly regulate microbial community structure by inhibiting the proliferation and activity of pathogenic bacterial species [28]. Therefore, it is hypothesized that the observed decrease in intestinal microbiota α-diversity may be attributed to the inhibition of symbiotic pathogenic bacterial survival. Firmicutes, as a core phylum within the intestinal microbiota, are pivotal in shaping microbial composition and substantially affect host physiological health. Studies have shown that SCFAs produced by Firmicutes can inhibit pathogen growth, suppress pro-inflammatory factors (like *tnf-α* and *il-6*) expressions, enhance intestinal epithelial cells’ tight junctions, stabilize the intestinal environment, and alleviate inflammatory bowel diseases [29]. Research on rats has shown that an HF diet increases *Actinobacteria* abundance in intestinal microbiota and significant lipid metabolism dysfunction [30]. Moreover, the pathogenic bacterial genus *Bosea* has been identified as a characteristic microbiota in the mice's intestines under an HF diet [31]. The present study revealed that RES supplementation in an HF diet significantly elevated the relative abundance of Firmicutes, decreased Actinobacteria, and altered the dominant status of *Bosea* within the intestinal microbiota of juveniles *M. amblycephala* under HF dietary conditions. These findings indicate that RES positively regulates intestinal microbial homeostasis and contributes to preserving intestinal function.

In the case of physical barriers, intestinal crypt depth and villus length are important indicators for assessing intestinal structural integrity. It is reported that RES supplementation in the HF diet significantly increased the intestinal villus length in rats, thus ameliorating HF diet-induced intestinal structural damage [32]. In the current study, RES addition significantly elevated intestinal villus length and muscle thickness of juvenile *M. amblycephala*, demonstrating its capacity to repair HF diet-induced intestinal structural damage. Moreover, epithelial tight junctions represent a basic structural component of the intestinal physical barrier, with junctional proteins such as Claudin, JAM, and Occludin serving crucial roles in maintaining intercellular cohesion and barrier functionality [33]. Dietary intake of HF regimens has been associated with elevated levels of intestinal lipopolysaccharide (LPS), which impairs the transcriptional expression of genes encoding tight junction-associated proteins [34]. Luo et al. [35] reported that RES administration effectively reversed LPS-induced downregulation of *zo-1*, *claudin-1*, and *occludin* genes expression in HT-29 cells, thus maintaining tight junction integrity and preventing epithelial barrier disruption. The current findings align with these observations; expression levels of *zo1*, *jam2*, and *claudin41* in juveniles *M. amblycephala* intestinal tissues in the HF-RES group (HF + 0.06% RES) exceeded those in the HF group, further confirming the RES role in enhancing intestinal barrier function under the HF diet.

It is reported that HF diets induced oxidative damage in intestinal tissues by generating reactive oxygen species (ROS) through fatty acid oxidation, depleting non-enzymatic antioxidants like reduced glutathione (GSH), and increasing LPO and MDA, thus disrupting tight junctions, enhancing intestinal permeability, and impairing barrier function in mice. However, RES treatment significantly increased intestinal GSH levels, reduced LPO and MDA concentrations, and ameliorated the mice’s oxidative stress [36]. Consistent with previous findings, RES supplementation counteracted the diet-induced reduction in GSH levels and the elevation of LPO and MDA concentrations, thus enhancing the antioxidant potential of the intestinal tract in juveniles *M. amblycephala*. Intestinal barrier dysfunction can trigger inflammation and metabolic disorders. At the molecular level, RES has been demonstrated to attenuate HF diet-induced inflammation by regulating multiple intracellular signaling pathways. Among these, inhibition of the TLR4/NF-κB signaling cascade (due to the lower secretion of pro-inflammatory mediators, i.e., IL-1β) is widely recognized as a main mechanism underlying its anti-inflammatory effects [22]. Similarly, the current findings found that in comparison with the HF group, supplementing RES significantly downregulated *tlr4*, *nf-κb,* and *il-1β* gene levels of juveniles *M. amblycephala* intestinal tracts.

A recent study has shown that RES, functioning as a methyl donor, positively affects lipid metabolic disturbances in HF diet-fed mice, primarily through the modulation of mRNA m^6^A modification levels [12]. These findings offer a valuable framework for elucidating the molecular mechanisms by which RES may alleviate lipid metabolism abnormalities in juvenile *M. amblycephala* by regulating intestinal function. Moreover, m^6^A methylation, recognized as the most prevalent epigenetic modification of RNA, is known to regulate various biological processes. The present study employed MeRIP-seq to identify 18,314 and 16,029 m^6^A peaks in the HF and HF-RES groups, respectively. These methylation peaks were predominantly localized within the 5′UTRs, CDS, and 3′UTRs, consistent with the m^6^A distribution patterns previously reported by Dominissini et al. [37]. It has been observed that m^6^A modifications frequently occur at consensus “RRACH” motifs, reflecting sequence specificity [37]. In the current analysis, similar motif patterns were detected in both HF and HF-RES groups, suggesting a potential role of m^6^A modification in mediating the regulatory effects of RES on intestinal physiology.

Several studies indicate that RES can achieve comprehensive regulation of intestinal function through multiple signaling pathways, including NF-κB, MAPK, and TGF-beta, from anti-inflammatory, anti-oxidation, barrier function enhancement, and other ways [38]. Combined examination of RNA-seq and MeRIP-seq datasets revealed that genes showing differential expressions at both mRNA and m^6^A methylation levels were primarily enriched in signaling pathways (TGF-beta, protein processing in endoplasmic reticulum, autophagy–animal, MAPK, and gap junction). Furthermore, the presented data highlighted significant enrichment of endoplasmic reticulum stress (ERS)-related pathways within the identified m^6^A methylation peaks, suggesting that m^6^A modifications may play a pivotal role in RES-mediated regulation of intestinal function, potentially through mechanisms intricately related to ERS modulation. The endoplasmic reticulum is the site of protein folding and maturation in the eukaryotic cell. Studies have shown that prolonged HF diet consumption can cause ERS in the intestinal tissues of *Micropterus salmoides*. ERS can mediate protein homeostasis imbalance by activating UPR and serve as an upstream regulatory hub connecting inflammatory responses and intestinal barrier damage [39]. RES has been observed to alleviate ERS via multiple mechanisms, including modulating UPR, balancing autophagy and apoptosis, and regulating calcium homeostasis through PERK/ATF4/CHOP [40,41].

Sequencing analysis revealed that RES supplementation in the context of an HF diet significantly increased both m^6^A methylation levels and the transcriptional expression of *hsp70*. As a key member of the heat shock protein family, HSP70 plays a pivotal role in regulating the unfolded protein response (UPR), thus preserving endoplasmic reticulum (ER) homeostasis. This is achieved through its chaperone activity by interacting with ERS sensors, such as ATF6, or by reducing mTOR-dependent suppression of autophagy [42]. Under conditions where ER stress exceeds cellular adaptive capacity, HSP70 stabilizes Bcl-2 family proteins by inhibiting CHOP-driven apoptotic signaling, ultimately attenuating ERS-induced apoptotic pathways [43]. Studies indicate that under acute heat shock stress, METTL3-mediated m^6^A modification recruits nuclear-localized YTHDF2 to protect HSP70 mRNA from erasure by the demethylase FTO, thereby significantly enhancing HSP70 protein synthesis efficiency and alleviating heat-induced damage [44]. In the present study, the expression of *hsp70* was quantified using qRT-PCR, along with the evaluation of genes involved in m^6^A RNA methylation (*mettl3*, *fto*, *alkbh5*, and *ythdf2*) and ER stress-related markers (*mtor*, *atf6*, *chop*, and *bcl-2*). The analysis indicated that adding RES to an HF diet significantly upregulated the *mettl3*, *hsp70*, *atf6,* and *bcl-2* expressions, and downregulated *fto*, *alkbh5*, *mtor,* and *chop*. These findings suggested that RES may enhance the m^6^A methylation level of *hsp70* by modulating key methylation-related enzymes, including *mettl3*, *fto*, and *alkbh5*, thus promoting *hsp70* gene expression. Thereby alleviating HF diet-induced ERS and modulating intestinal function of juvenile *M. amblycephala* by regulating the UPR (upregulating *atf6*) or inhibiting apoptosis (downregulating *mtor* and *chop*).

Studies have shown that ERS-induced disruption to intestinal barrier function allows intestinal LPS and free fatty acids to enter the liver via portal vein circulation, thus inhibiting fatty acid β-oxidation and bile acid synthesis, exacerbating steatosis and contributing to the intestine–liver axis lipid metabolism disorders [45,46]. In this study, intergroup Pearson correlation analysis revealed a negative association between the gene level of intestinal *hsp70* and plasma ALT level, a positive association between intestinal *atf6* gene level and hepatic *ppar-α* gene expression, and positive correlations between intestinal *bcl-2* and hepatic *ppar-β* and *cyp7a1* expressions. Compared with the HF group, the HF-RES group depicted upregulated expressions of *atf6*, *bcl-2*, *ppar-α*, *ppar-β*, and *cyp7a1*, indicating that RES can promote hepatic fatty acid β-oxidation and bile acid synthesis by improving HF diet-induced ERS via m^6^A methylation, thus alleviating lipid metabolism disorders in *M. amblycephala*.

Mantel test correlation analysis revealed that intestinal microbiota is directly associated with ERS, tight junctions in intestinal epithelial cells, and inflammatory responses. Studies report that intestinal microbiota have the potential to regulate tight junction protein expression through metabolites such as SCFAs, and then maintain intestinal integrity. Microbial dysbiosis can induce ERS, disrupt tight junction structures, increase intestinal permeability, and exacerbate inflammatory responses [47,48]. The experimental results showed that the relative abundance of the opportunistic bacterium *Bosea* was positively associated with the expression of ERS-related gene *mtor* and the pro-inflammatory gene *tlr4*. RES supplementation significantly decreased the intestinal abundance of *Bosea* and the expression levels of *mtor* and *tlr4* in *M. amblycephala*. These observations suggested that the intestinal protective effects of RES may involve a favorable regulatory loop, characterized by restoring microbial homeostasis, attenuating ERS, preserving intestinal barrier integrity, and suppressing inflammatory signaling pathways.

## 4. Materials and Methods

### 4.1. Experimental Diets

Resveratrol (RES; HPLC ≥ 98%) was purchased from Beyotime Biotechnology Co., Ltd. (Shanghai, China). As per the methodology outlined by Yan [49], soybean oil was used as the dietary lipid source, while fishmeal, soybean meal, rapeseed meal, and cottonseed meal were the main protein sources for the preparation of five experimental diets. The diets included a conventional diet (CON group), an HF diet (HF group), and an HF diet enriched with 0.6 g/kg RES (HF + 0.06% RES group), 3 g/kg RES (HF + 0.3% RES group), or 6 g/kg RES (HF + 0.6% RES group). The composition of each diet is detailed in Table 2. All ingredients were finely ground, passed through a 60-mesh sieve, and weighed precisely according to the prescribed formulation. All components were homogenized, followed by the addition of water and soybean oil. The resulting mixture was extruded into 2 mm pellets using an F-26 (II) granulator (South China University of Technology, Guangzhou, China). After air-drying, the pellets were stored at −20 °C until further use.

### 4.2. Fish and Feeding Trial

An indoor system with recirculating water system with and temperature control was used for the aquaculture trial. Juveniles *M. amblycephala* were provided by *M. amblycephala* National Breeding Farm (Wuhan, China). A total of 225 4-month-old healthy fish (8 ± 0.5 g) were randomly divided into 15 glass tanks and divided into five dietary groups: CON, HF, HF + 0.06% RES, HF + 0.3% RES, and HF + 0.6% RES. Each group comprised three replicate tanks with a 15 fish per 350 L tank. For 8 weeks, the juveniles were fed to apparent satiety three times daily (07:00, 12:00, and 17:00). The feeding trial was conducted under natural light conditions, with water temperature maintained at 26–28 °C, ammonia nitrogen ≤ 0.05 mg/L, dissolved oxygen ≥ 6 mg/L, and pH 7.0–7.5.

### 4.3. Sample Collection

After feeding for 8 weeks, fish were fasted for 24 h and anesthetized using MS-222 (100 mg/L; Sigma, Saint Louis, MO, USA). The number and weight of fish in each tank were recorded to determine growth performance indices. Three fish per tank were randomly selected for blood collection via the caudal vein. Plasma was obtained by centrifugation at 4000 rpm for 10 min at 4 °C, and the supernatant was stored at −20 °C for later biochemical analysis. Liver, hind intestine, and hind intestinal contents were quickly harvested in duplicate, flash-frozen in liquid nitrogen, and stored at −80 °C. One set was allocated for hepatic and intestinal gene expression analysis, while the other was used for 16S rRNA sequencing of hind intestinal contents and MeRIP-seq of hind intestinal tissue. An additional three fish per tank were sampled to collect liver and hind intestine tissues; a portion of the tissue was stored at −20 °C for antioxidant enzyme activity analysis, and the remaining was fixed in 4% paraformaldehyde (PFA) for histopathological analysis using Nile Red and H&E staining.

### 4.4. Parameter Measurement

#### 4.4.1. Growth Performance Analysis

Specific growth rate (SGR, %/d) = 100 × (ln*W_t_* − ln*W*_0_)/*t*;

Weight gain rate (WGR, %) = 100 × (*W_t_* − *W*_0_)/*W*_0_;

Feed conversion ratio (FCR) = *F*/(*W_t_* − *W*_0_).

where

*W_t_* = final total weight of fish (g);

*W*_0_ = initial total weight of fish (g);

*t* = feeding duration (d);

*F* = total feed intake (air-dry basis, g).

#### 4.4.2. Biochemical Index Analysis

Plasma LDL, TC, and ALT levels were measured using a BS-400 Q2080 fully automated biochemical analyzer (Mindray, Shenzhen, China), employing commercial kits. The concentrations of GSH and MDA, and LPO activities in hind intestinal tissues were quantified using a microplate reader (Thermo Fisher, Cleveland, OH, USA). All examination kits were provided by the Nanjing Jiancheng Bioengineering Institute (Nanjing, China).

### 4.5. Histological and Fluorescence Staining

Hepatic tissues fixed in PFA were processed into frozen sections for Nile Red staining. Following the method of Shen et al. [50], sections were air-dried at room temperature, encircled using a hydrophobic barrier pen, and stained with Nile Red solution in the dark. After rinsing and drying, DAPI was used in the dark for nuclear staining. After final washing, sections were sealed using a fade-resistant mounting medium and examined under a fluorescence microscope. The red lipid deposition area (%) in hepatic tissue was quantified using ImageJ software (v1.8.0.112).

Based on Kamyab-Hesary et al. [51] PFA-fixed intestinal tissues were trimmed and embedded in paraffin after dehydration and waxing steps. Paraffin blocks were sectioned, deparaffinized with xylene, and rehydrated through a graded ethanol series. Staining was performed using Hematoxylin (nuclear) and Eosin (cytoplasmic) dyes. Slides were mounted and examined microscopically to assess histological morphology, followed by image acquisition.

### 4.6. Microbiota Analysis in Intestinal Contents

Four hind intestinal content samples were prepared for both the HF group and the HF + 0.06%RES group. Three of these were composed of an equal-quantity mixture of intestinal contents from three fish per replicate, respectively, while the remaining sample was a pooled mixture formed by combining equal volumes of these three mixtures. This pooled sample integrated intestinal contents from all nine fish within the treatment group. As described by Qian et al. [52], genomic DNA was extracted and evaluated for quality and concentration via 1% agarose gel electrophoresis. PCR was amplified with specific primers targeting the V4-V5 hypervariable region of the bacterial 16S rRNA gene. Following sequencing standards, PCR products were purified and pooled in defined ratios. Library sequencing was performed on the Illumina platform (PE250) (Illumina, San Diego, CA, USA). Optimized data were clustered into OTUs at 97% sequence identity, and the taxonomic assignment was executed using the RDP Classifier with a Bayesian algorithm. Microbial community composition was statistically analyzed [53].

### 4.7. MeRIP-seq

#### 4.7.1. RNA Extraction, Library Preparation, and Sequencing

According to previously reported procedures [54], the isolation of total RNA from juvenile *M. amblycephala* hind intestinal tissue samples of the HF and HF + 0.06%RES group was performed using TRIzol reagent (Invitrogen, Carlsbad, CA, USA). A ND-1000 spectrophotometer (NanoDrop Technologies, Wilmington, DE, USA) was used to assess the quality of the extracted RNA. Oligo (dT) magnetic beads were used to selectively capture Polyadenylated RNA via two rounds of purification. After being fragmented, a portion of the RNA was pre-mixed with magnetic beads and an m^6^A-specific antibody for immunoprecipitation (IP), while the remaining portion served as the input sample and underwent direct library construction. Before the second strand synthesis, the IP product was reverse-transcribed into cDNA. cDNA fragments were size-selected and purified using magnetic beads. Sequencing libraries were constructed via PCR amplification and sequenced on Illumina Novaseq^TM^ 6000 (PE150) (Illumina, San Diego, CA, USA).

#### 4.7.2. Bioinformatics Analysis

After quality control, clean reads from all IP and input samples were aligned to the *M. amblycephala* reference genome using HISAT2 (http://daehwankimlab.github.io/hisat2, accessed on 2 September 2024) [55]. The R package (version 3.3) exomePeak was used for differential peak analysis of BAM files (https://www.bioconductor.org/packages/3.3/bioc/html/exomePeak.html, accessed on 13 September 2024). Peak annotation was conducted using ANNOVAR (Gencode v41 Basic collection) (http://www.openbioinformatics.org/annovar/, accessed on 17 September 2024) and motif enrichment analysis was performed using HOMER (http://homer.ucsd.edu/homer/motif, accessed on 21 September 2024) [56]. Gene assembly and quantification were conducted via StringTie (https://ccb.jhu.edu/software/stringtie, accessed on 8 October 2024) and gene expression levels were computed using the FPKM method. DEGs were identified using the R package edgeR, with selection criteria set at |log_2_(fold change)| ≥ 1 and *p* < 0.05 [57].

### 4.8. Real-Time PCR Analysis

After the procedure of the previous research [52], the RNAiso Plus Kit (Takara, Daliang, China) was used to isolate total RNA from the hind intestines of *M. amblycephala*. RNA concentration and quality were assessed by measuring OD 260/280 ratios (1.8–2.1). Target gene expression was quantified using cDNA, which was synthesized by the extracted RNA, as the template via qRT-PCR. The reference gene *β-actin* was used to calculate relative gene expression through the 2^−ΔΔCT^ method. The sequences of primers are provided in Table 3, and primers were composed in Sangon Biotech (Shanghai, China).

### 4.9. Statistical Analysis

Statistical analyses were performed using SPSS 25.0 software. The experimental data were examined to determine if they followed a normal distribution via the Shapiro–Wilk test, and confirmed to meet the requirements of variance homogeneity via Levene’s test. Statistical comparisons were conducted using one-way ANOVA followed by Duncan’s test or an Independent *t*-test. Data are presented as mean ± standard error mean (SEM). Statistical significance was attributed to the intergroup differences when *p* < 0.05.

## 5. Conclusions

In conclusion, this study elucidates the regulatory mechanisms by which RES modulates intestinal function and mitigates lipid metabolism disorders in high-fat diet-fed juveniles *M. amblycephala*, as revealed through integrated multi-omics analyses. Dietary RES supplementation contributed to maintaining the homeostasis of the intestine–liver axis and reducing hepatic lipid accumulation by stabilizing intestinal microbiota composition, modulating m^6^A methylation levels, alleviating endoplasmic reticulum stress, restoring intestinal barrier integrity, and suppressing inflammatory signaling pathways. These findings not only provide a theoretical basis for the application of RES as a functional nutraceutical in aquaculture, and highlight the pivotal role of epigenetic regulation in maintaining intestinal health and metabolic balance in aquatic species.

## Figures and Tables

**Table 1:** All 20 m^6^A peaks with the most significant methylation differences.

Gene	GeneID	DiffModLog_2_FC ^1^	*p*-Value	Chromsome	Start	End	Peak-Length	Region
*nynrin*	LOC125243639	6.44	0.00	NC_063056.1	7,889,886	7,890,097	212	3′UTR
*histone H3. v1*	LOC125250419	5.66	0.00	NC_063060.1	41,658,950	41,662,658	3709	3′UTR
*neu4*	LOC125252118	5.66	0.00	NC_063061.1	11,575,766	11,576,660	895	exonic
*ceacam1*	LOC125263488	5.25	0.03	NC_063045.1	9,519,250	9,519,431	182	exonic
*otud7a*	otud7a	5.09	0.04	NC_063058.1	9,780,589	9,780,739	151	3′UTR
*irf4*	LOC125253556	5.04	0.00	NC_063061.1	7,605,408	7,612,562	7155	3′UTR
*trim39*	LOC125263520	−4.92	0.00	NC_063045.1	10,315,588	10,315,953	366	exonic
*ttc38*	LOC125253910	−4.30	0.00	NC_063062.1	22,714,721	22,715,566	846	5′UTR
*znf184*	LOC125248635	4.06	0.01	NC_063059.1	40,038,026	40,049,733	354	3′UTR
*slit1a*	slit1a	4.03	0.00	NC_063053.1	37,082,927	37,183,886	691	3′UTR
*slc2a12*	slc2a12	3.81	0.02	NC_063067.1	15,936,237	15,936,508	272	exonic
*zfy1*	LOC125266323	3.64	0.02	NC_063047.1	3,706,888	3,707,277	390	exonic
*bmp1a*	bmp1a	3.64	0.03	NC_063055.1	35,350,248	35,350,428	181	3′UTR
*cyyr1*	cyyr1	3.54	0.01	NC_063050.1	56,166,843	56,167,050	208	5′UTR
*mtres1*	mtres1	3.42	0.03	NC_063053.1	20,679,502	20,682,293	2792	5′UTR
LOC125253164	LOC125253164	3.39	0.01	NC_063061.1	32,536,181	32,536,362	182	exonic
*nfe2l1b*	nfe2l1b	3.38	0.02	NC_063063.1	20,348,636	20,348,876	241	exonic
*znf692*	znf692	3.32	0.03	NC_063055.1	13,164,878	13,165,057	180	exonic
LOC125260582	LOC125260582	−3.30	0.00	NC_063067.1	4,134,964	4,135,136	173	exonic
*ri*	LOC125250829	−3.14	0.02	NC_063060.1	47,937,450	47,937,624	175	3′UTR

^1^ The log_2_-transformed fold change in differential methylation peaks between the HF-RES and HF groups.

**Table 2:** Basic feed formulation and nutrient composition for all experiment groups (%, dry basis).

Ingredient	CON	HF	HF + 0.06%RES	HF + 0.3%RES	HF + 0.6%RES
Fishmeal ^1^	6.00	6.00	6.00	6.00	6.00
Soybean meal (46%) ^1^	30.00	30.00	30.00	30.00	30.00
Rapeseed meal ^1^	12.00	12.00	12.00	12.00	12.00
Cottonseed meal ^1^	10.00	10.00	10.00	10.00	10.00
Cottonseed protein concentrate^1^	4.00	4.00	4.00	4.00	4.00
Wheat flour ^1^	18.00	10.00	10.00	10.00	10.00
Rice bran ^1^	6.00	7.00	7.00	7.00	7.00
Soybean oil ^2^	4.50	11.50	11.50	11.50	11.50
Calcium dihydrogen phosphate ^1^	2.00	2.00	2.00	2.00	2.00
Mineral premix ^1^	0.50	0.50	0.50	0.50	0.50
Vitamin premix ^1^	0.50	0.50	0.50	0.50	0.50
Vitamin C ^1^	1.00	1.00	1.00	1.00	1.00
Choline chloride ^1^	0.50	0.50	0.50	0.50	0.50
Microcrystalline cellulose ^1^	5.00	5.00	4.94	4.70	4.40
RES	0	0	0.06	0.30	0.60
Total	100.00	100.00	100	100	100
Nutrition composition (air dry basis)					
Crude protein, %	32.75	31.79	31.94	32.03	31.68
Crude fat, %	7.70	14.76	14.46	14.35	14.58
Gross energy (KJ/g)	16.23	15.01	14.73	15.16	14.91

^1^ Provided by Beijing Dabeinong Technology Group Co., Ltd. (Shanghai, China). ^2^ Provided by Fulinmen Commodity Soybean Oil, purchased from the local RT-mart supermarket (Wuxi, China).

**Table 3:** Sequences of primers used for qRT-PCR.

Gene	Primer Sequence (5′-3′)	Product Length (bps)	Accession No.
*tlr4*	F: GAATGCTGGACAAGGACAGGA	102	XM_048204248.1
	R: GTGATAGGAAGACTGCTGGGA		
*nf-κb*	F: AGTCCGATCCATCCGCACTA	85	XM_048176853.1
	R: ACTGGAGCCGGTCATTTCAG		
*il-1β*	F: ACCAGCACGACCTTGCAGTG	174	XM_048181166.1
	R: CTGGGATGCATTCGGTTTGA		
*jam2*	F: CCTCCGTGGTGTTACACAGA	105	XM_048193479.1
	R: AGCACATTGAGGGTGACGAT		
*zo1*	F: CCTCTGGTGATGTGTGGTCC	75	XM_048184358.1
	R: AGACGCACAATGAGGTAGGC		
*claudin41*	F: TTGTGATTGGGATCCTGGGC	84	XM_048167602.1
	R: TGGTTTTGGAGCTCTCGTCC		
*ppar-α*	F: GTGCCAATACTGTCGCTTTCAG	104	XM_048158021.1
	R: CCGCCTTTAACCTCAGCTTCT		
*ppar-β*	F: CATCCTCACGGGCAAGAC	150	XM_048209548.1
	R: TGGCAGCGGTAGAAGACA		
*lpl*	F: TCTGATGGGATCTGGCAC	85	XM_048164066.1
	R: GTTTCTGGATTTGGGTCG		
*cyp7a1*	F: TTTCCGTCAGACGCTTCAGG	123	XM_048186424.1
	R: CCCTTCTTCAAGCCAGTCGT		
*hsp70*	F: CCAGGTGTACGAGGGAGAGA	113	XM_048209656.1
	R: AGGTCACTTCAATCTGCGGG		
*atf6*	F: CGATCAGGATGGAGAGTGGGATA	108.30	XM_048207041.1
	R: AGGGCTACTCCACAATGGGT		
*chop*	F: ATGTGGTGCAGAGTTGGAGG	108.20	XM_048198700.1
	R: CACATCCAGAAACTCGGGCT		
*bcl-2*	F: CGTCTACCTGGACAACCACA	106.50	XM_048179299.1
	R: GCGTTTCTGTGCAATGAGTG		
*mtor*	F: ACGGTCTCTACTCTGCCAGT	106.30	XM_048206869.1
	R: ACCAGGGGGCATAAAACTCG		
*mettl3*	F: GTTTGCAGTGGTGATGGCTG	103.20	XM_048185876.1
	R: TTTCGTGTGCCTGGAGACAG		
*fto*	F: GATTCTGCAGCTGGTGGACT	103.50	XM_048184627.1
	R: GTCTGTCTGTGCTGCTGTCT		
*alkbh5*	F: GATCGATGAGGTGGTTGCCA	109.20	XM_048197746.1
	R: TACGTGTAGCCCTCTCCGAA		
*ythdf2*	F: GGACAAGTGGAAGGGACGTT	100.40	XM_048191909.1
	R: TCCAGTGGAACCTCCTGAGT		
*β-actin*	F: TCGTCCACCGCAAATGCTTCTA	152	AY170122.2
	R: CCGTCACCTTCACCGTTCCAGT		

## Data Availability

The authors confirm that the data supporting the findings of this study are available within the manuscript and tables.

## Supplementary Materials

The following supporting information can be downloaded at: https://www.mdpi.com/article/10.3390/ijms26178587/s1.
